# 

**Published:** 1885-04

**Authors:** 


					﻿WhVe Number,
CCLXXV.
APRIL, 1885.
Number 9,
Volume XXIV.
THE
B U F F
Medical and SumS£2gurnal.
EDITORS:
THOS. I.OTHRQP, M. D»,	A. R. DAVIDSON, M.
Published by the Medical Journal Association.
No. B WEST CHIPPEWA ST.
Entered at the Post-Office at Buffalo, N. Y., as Second-class Mail Matter.
				

